# Opinions of key stakeholders on alternative interventions for malaria control and elimination in Tanzania

**DOI:** 10.1186/s12936-020-03239-z

**Published:** 2020-04-23

**Authors:** Marceline F. Finda, Nicola Christofides, Javier Lezaun, Brian Tarimo, Prosper Chaki, Ann H. Kelly, Ntuli Kapologwe, Paul Kazyoba, Basiliana Emidi, Fredros O. Okumu

**Affiliations:** 1 Environmental Health and Ecological Sciences Department, Ifakara Health Institute, P. O. Box 53, Ifakara, Tanzania; 2 School of Public Health, Faculty of Health Sciences, University of the Witwatersrand, 1 Smuts Avenue, Braamfontein, 2000 South Africa; 3 Institute for Science, Innovation and Society, School of Anthropology and Museum Ethnography, University of Oxford, Oxford, UK; 4 Department of Global Health and Social Medicine, King’s College, London, UK; 5 President’s Office, Regional Administration and Local Government, P. O Box 1923, Dodoma, Tanzania; 6 National Institute for Medical Research, 3 Barack Obama Drive, Dar es Salaam, Tanzania; 7 National Malaria Control Programme, P. O. Box 743, Dodoma, Tanzania; 8 Institute of Biodiversity, Animal Health and Comparative Medicine, University of Glasgow, Glasgow, G12 8QQ UK; 9 School of Life Science and Bioengineering, The Nelson Mandela African Institution of Science and Technology, P. O. Box 447, Arusha, Tanzania

**Keywords:** Malaria control, Malaria elimination, Stakeholders, Tanzania, Opinion, Alternative interventions

## Abstract

**Background:**

Malaria control in Tanzania currently relies primarily on long-lasting insecticidal nets and indoor residual spraying, alongside effective case management and behaviour change communication. This study explored opinions of key stakeholders on the national progress towards malaria elimination, the potential of currently available vector control interventions in helping achieve elimination by 2030, and the need for alternative interventions that could be used to supplement malaria elimination efforts in Tanzania.

**Methods:**

In this exploratory qualitative study, Focus group discussions were held with policy-makers, regulators, research scientists and community members. Malaria control interventions discussed were: (a) improved housing, (b) larval source management, (c) mass drug administration (MDA) with ivermectin to reduce vector densities, (d) release of modified mosquitoes, including genetically modified or irradiated mosquitoes, (e) targeted spraying of mosquito swarms, and (f) spatial repellents.

**Results:**

Larval source management and spatial repellents were widely supported across all stakeholder groups, while insecticide-spraying of mosquito swarms was the least preferred. Support for MDA with ivermectin was high among policy makers, regulators and research scientists, but encountered opposition among community members, who instead expressed strong support for programmes to improve housing for poor people in high transmission areas. Policy makers, however, challenged the idea of government-supported housing improvement due to its perceived high costs. Techniques of mosquito modification, specifically those involving gene drives, were viewed positively by community members, policy makers and regulators, but encountered a high degree of scepticism among scientists. Overall, policy-makers, regulators and community members trusted scientists to provide appropriate advice for decision-making.

**Conclusion:**

Stakeholder opinions regarding alternative malaria interventions were divergent except for larval source management and spatial repellents, for which there was universal support. MDA with ivermectin, housing improvement and modified mosquitoes were also widely supported, though each faced concerns from at least one stakeholder group. While policy-makers, regulators and community members all noted their reliance on scientists to make informed decisions, their reasoning on the benefits and disadvantages of specific interventions included factors beyond technical efficiency. This study suggests the need to encourage and strengthen dialogue between research scientists, policy makers, regulators and communities regarding new interventions.

## Background

Morbidity and mortality due to malaria has significantly declined worldwide over the past two decades, most significantly in sub Saharan Africa [1, 2]. Between 2000 and 2017 the number of malaria cases recorded in the region has decreased by 41%, and mortality by 62%, a success attributable to both public health efforts and improvements in socioeconomic conditions [1]. In Tanzania, malaria prevalence has gone down by more than 50% over the past decade, from 18% in 2008 to just 7.3% in 2017, mainly as a result of near universal coverage with long-lasting insecticide-treated bed nets (LLINs), indoor residual sprays (IRS), reliable and affordable diagnosis and treatment, and improved livelihoods [3].

The interventions behind this success in malaria control are however rapidly reaching their limits, as malaria continues to persist. A significant slowdown in the decline of malaria cases and deaths has been observed over the past decade; between 2010 and 2018, the rate of malaria cases and deaths have only declined by 22% and 30%, respectively [2]. Mosquito resistance to insecticides used in indoor residual spraying and bed nets is now widely documented worldwide [4, 5]. An increasing disposition of malaria vector to bite during the early-evening hours and the early morning, and to do so outdoor, is also well documented [6, 7], threatening the success of the major interventions for malaria control. *Plasmodium* resistance to the commonly used drugs is also increasingly evident across Africa and Asia [8, 9], further threatening the progress.

In 2015, the World Health Assembly adapted the Global Technical Strategy for Malaria 2016–2030, which aimed to provide a framework to reduce malaria incidence and mortality by 90% worldwide by 2030, and to eliminate malaria in 35 countries by the same year [10]. Tanzania is one of the countries currently pursuing malaria elimination by 2030, building on the significant gains achieved since the late 1990s [11]. To achieve malaria elimination, the National Malaria Control Programme has adopted a strategy to ensure adequate coverage of vector control interventions, primarily the use of LLINs and IRS [11]. The strategy also includes improved malaria diagnosis and case management, as well as roll-out of new complementary interventions where there is sufficient local evidence for impact [11].

Several complementary vector control interventions are currently being discussed as possible candidates to accelerate the malaria elimination efforts [12]. Examples include: (a) larval source management (LSM), including larviciding and environmental management [13, 14], (b) topical repellents for personal protection [15, 16], (c) mass drug administration with endectocides such as ivermectin [17, 18], (d) use of mosquito modification techniques, either to suppress or replace vector populations [19, 20], (e) outdoor targeting of male mosquitoes through insecticide-spraying of mosquito swarms [21–23], (f) housing improvement measures such as better window screening and improved house designs [24–26], (g) spatial repellents able to protect multiple individuals over wide areas [27, 28], (h) attractive toxic sugar baits targeting sugar-seeking mosquitoes [29, 30], and (i) mosquito-killing fungal spores and toxins [31, 32].

Unfortunately, most of these interventions are not ready for deployment at scale; significant investments, as well as strong multi-sectorial collaborations will be needed to complete their development and evaluation. To ensure that these potential alternative interventions meet user needs and are sustainable, it is crucial to consider, early on in their development, the views and opinions of key stakeholders. This study sought to explore opinions of key stakeholders regarding Tanzania’s progress towards malaria elimination, the potential of currently available vector control interventions to achieve elimination by 2030, and the potential and acceptability of additional vector control interventions that could supplement current elimination efforts. This study is a part of a larger investigation seeking to assess the awareness and perceptions of alternative strategies for malaria control and elimination in Tanzania, and to design appropriate pathways for the development of new intervention packages.

## Methods

### Study site and stakeholder selection

This study was done in Tanzania between December 2018 and May 2019, and involved four groups of stakeholders: (a) policy-makers, (b) regulators, (c) research scientists, and (d) community members. The stakeholders were all involved either directly or indirectly in malaria control in Tanzania.

Research scientists were selected from two leading research institutes in the country: Ifakara Health Institute (IHI), and National Institute for Medical Research (NIMR). The group included entomologists, economists, health systems and policy researchers, molecular biologists and ethicists involved in the design of malaria control strategies in Tanzania. The group of policy-makers included senior officials from government ministries in Dodoma, Tanzania’s administrative capital, all of them with direct or indirect influence on malaria control activities. The government ministries represented were: (a) Ministry of Health, Community Development, Gender, Elderly and Children, (b) Ministry of Education and Vocational Training, (c) Ministry of Agriculture, (d) Ministry of Livestock and Fisheries Development, (e) Ministry of Water and Irrigation, (f) Ministry of Housing and Infrastructure and (g) President’s Office-Regional Administration and Local Government. The group of regulators included officials from the Tanzania Medicines & Medical Devices Authority, the Tanzania Commission for Science and Technology, and the National Environmental Management Committee.

Lastly, community members were comprised of local community leaders drawn from 10 rural and urban wards in Ulanga and Kilombero districts in the Kilombero valley, in south-eastern Tanzania. Residents in the area are mostly subsistence farmers, pastoralists or small business owners [33, 34]. Malaria prevalence in these districts is highly heterogeneous, ranging from < 1% in the urban and peri-urban sites to > 40% in some of the villages (Swai et al., unpublished). Transmission intensities are also highly diverse, varying from less than 1 to ~ 20 infectious bites per person per year [33, 34].

### Study procedures and interventions evaluated

This was an exploratory qualitative study; moderated Focus group discussions (FGDs) [35] were used to explore opinions of the stakeholders on the suitability and potential of alternative interventions. The alternatives discussed were: (a) improved housing, (b) larval source management, (c) mass drug administration (MDA) with ivermectin to reduce vector densities, (d) release of modified mosquitoes, including genetically modified strains, (e) targeted spraying of mosquito swarms, and (f) spatial repellents. These interventions were selected because of their pertinence for policy discussions, whether it is being considered for large scale implementation for malaria vector control in the country, undergoing large clinical trials in the country, or gaining interest worldwide as potential tools to help achieve malaria elimination. Table 1 shows summaries of these interventions, including some evidence on their potential.

**Table 1:** Descriptions of alternative interventions to complement current malaria control and elimination efforts, as discussed with key stakeholders in Tanzania

Intervention	Description
Improved housing	House improvement as malaria control intervention involves mosquito-proofing houses to limit mosquito entrance into the house [26, 37]. General housing improvement was a key factor in the elimination of malaria in developed countries [24]. In developing countries, simple modifications like screening windows and doors and closing eave spaces have resulted in some cases, in a 50% decline in entomological inoculation rates [38]. In Tanzania for example, housing improvement was linked to significant historical declines of malaria in Dar es Salaam [39], and was likely a major factor in more than 99% decline in malaria in Ifakara town, the main town in the area of our study [33]
Larval source management	Larval source management (LSM) refers to environmental manipulations to target mosquito larval habitats [13]. LSM can include the use of larvicides as well as environmental management methods [13, 14, 40]. In Tanzania, large-scale larviciding resulted in 21% reduction in malaria prevalence in Dar es Salaam between 2006 and 2008 [41]. The Tanzanian government is currently conducting targeted larviciding in urban and rural settings as a means to reduce malaria incidence and speed up the elimination agenda [42]
Mass drug administration of ivermectin	Ivermectin is an anti-helminthic drug commonly used to control parasitic nematodes in humans and animals [43]. It has been extensively used in mass campaigns for the elimination of lymphatic filariasis and onchocerciasis in Tanzania [44, 45]. Ivermectin is currently being evaluated as a malaria control tool, since it significantly reduces female mosquito fecundity and survival when mosquitoes blood-feed on hosts that have taken the drug [18, 43, 46]
Targeted spraying of mosquito swarms	Male mosquitoes aggregate in swarms as they compete for attention of female mosquitoes searching for mating partners [47]. Swarms usually occur at approximately the same time, usually at sunset, and repeatedly at same locations throughout the year [47]. Studies done in Burkina Faso and Tanzania have shown that *Anopheles* mosquito swarms can be located and targeted, and are effective in reducing overall mosquito density [21–23]
Modified mosquitoes	This intervention refers to alterations of mosquito genes or physiology for the purpose of reducing their competence in diseases transmission. The modified mosquitoes are released into the environment so that they can interbreed with the wild mosquitoes and, depending on the trait they carry, either reduce the density of malaria vectors or replace its population with mosquitoes unable to transmit the pathogen. Interventions currently under study include Sterile Insect-technique, which relies on irradiation of mosquitoes to make them sterile [48], genetic modification of mosquitoes to introduce sterility or other disadvantageous traits [49], and use of gene drive systems to spread novel traits (e.g. lethality or refractoriness to pathogen transmission) in mosquito populations [19, 50]. While the technology has never been integrated into a malaria control programme, laboratory studies, mathematical models and preliminary field trials indicate its potential [51]
Spatial repellents	Spatial repellents prevent host-seeking mosquitoes from entering certain areas, thus limiting contact between humans and mosquitoes [27]. SP can be based on a variety of botanical products and chemical compounds, such as citronella, transfluthrin and metofluthrin [27, 52]. They can be delivered in different formats, such as mosquito coils, repellent-treated clothing, repellent sandals (Finda et al. unpublished), kerosene lamps [53], and eave ribbons [27, 28, 54]. Compared to widely available topical repellents, some SP can provide long-lasting repellency, requiring minimal participation from the users

A total of eight focus group discussion sessions, two per stakeholder group, were conducted, each with 6–10 participants. During the FGDs with community members, men and women were separated to maximize the participation of women [35]. This separation was considered unnecessary for the other stakeholder groups. To avoid framing the discussions too narrowly, a semi-structured discussion guide was used. Participants were first asked open-ended questions about their opinions on the country’s progress towards malaria elimination, their views on the effectiveness of current malaria control interventions, and the need for alternative interventions for malaria control. The facilitator then presented a brief overview of the alternative interventions for malaria elimination, by way of PowerPoint slides. The presentations were delivered in English with the expert groups, but the language was adapted to Swahili (the main language spoken in Tanzania) for the two FGDs with community members. Participants were given time to ask questions following the presentation of each intervention, and when they were satisfied with the answers the discussions about that specific interventions began. The FGD sessions lasted 120–150 min each and were audio-recorded with participants’ consent. Additionally, detailed notes were taken during the discussions.

Participants from each stakeholder group were purposively selected with help from their institutional leaders. It was important that stakeholders with expertise in malaria control were identified. With regards to the experts, invitation letters were sent to heads of institutions were the participants were based, and these heads then recommended staff members for the discussions. With the community members, ten wards were randomly selected in the Kilombero Valley in south-eastern Tanzania, and invitation letters were sent to ward leaders to recommend one male and one female community leaders to participate in the discussions.

The discussions were facilitated by two research scientists from Ifakara Health Institute, both of whom have extensive knowledge of malaria control. While the scientists were known to some of the participants because of their work, there were no subordinate relationships between facilitators and participants. FGDs with research scientists and policy makers were undertaken in their respective institutes. In the case of community members and regulators, the discussions were done centrally at Ifakara Health Institute offices. The feedback sessions were also done at Ifakara Health Institute.

### Data processing and analysis

Audio recordings of the FGDs were transcribed immediately following the discussions, then translated from Swahili to English when needed. Field notes were incorporated in the written transcripts as additional data. The final transcripts were reviewed in detail then imported to Nvivo 12 Plus software [36] for further processing and analysis. Deductive analysis was used to categorize codes based on the FGD guide, which explored participants’ opinions on: (a) the country’s progress towards malaria elimination, (b) potential of current interventions for malaria elimination, (c) need for alternative approaches and techniques to support elimination efforts, (d) potential of the alternative interventions, and (e) their potential applications as complementary interventions in the efforts towards the 2030 malaria elimination target. Preliminary findings of the study were presented back to the participants. Quotes from participants were used to support the themes.

## Results

Altogether, 60 people participated in the FGD discussions from across the stakeholder groups, 33 of whom were males and 27 females. Demographic characteristics of the FGD participants are presented on Table 2. Results on the opinions of key stakeholders of malaria elimination in Tanzania are presented based on the FGD guide points listed on the “Data processing and analysis” section.

**Table 2:** Gender distribution of the participants of Focus group discussions

Stakeholder group	Males	Females	Total
Community members	8	8	16
Policy makers	7	8	15
Regulators	7	7	14
Research scientists	11	4	15

### Opinions on progress towards malaria elimination in Tanzania

Research scientists, regulators and policy makers discussed the progress made by Tanzania towards malaria elimination in terms of declining rates of malaria prevalence. Community members in contrast, discussed the progress in terms of their daily life experiences.

Two major arguments emerged in relation to this issue across the stakeholder groups. On the one hand, it was agreed that the country had made good progress and was on the right track. On the other hand, it was similarly noted that the progress was too slow and inadequate for elimination by 2030 as planned. Participants who emphasized that the country was on track referred to the significant reduction in malaria prevalence over the past decade, noting that malaria has reduced by more than 50% since 2000. As one policy maker stated:

“*Of course we have come far from when prevalence was as high as 20% in the whole country. Back then, when you look at the map, it was all red, all red I tell you. There was malaria everywhere. But now you can see quite a lot of places that have prevalence of less than 1%, so when I see that I know that we are doing well.”* (Policy-maker; female).

For community members, their idea of progress was informed mostly by their lived experiences. They noted, for example, that the frequency and severity of malaria attacks has greatly declined over the years. Unlike in the past, when malaria infections were very frequent, several months could now go by without their children getting sick. And when they did get sick, it was likely not to be malaria. As one participant said:

“*Ten years back there was a lot of malaria. During that time, every time you did not feel well and went to the hospital you would be told that you have malaria. Kids were getting sick very often. But now we can go for even six months without our children getting sick or needing to go to the hospital. And when we do go we hear about other diseases, like urinary tract infections or typhoid. So then I know that malaria is not a big disease like it used to be.”* (Community members; female).

Some participants, particularly policy makers and research scientists asked for caution, noting that, while there has been significant progress, it was nevertheless too slow and did not reflect the amount of effort that the country was putting into place. They also noted that the decline in malaria prevalence was not uniform across the country. As one policy maker reported:

*“I think we are doing well, but not as well as I would like. As a country we have put a great deal of efforts to finish off this disease, but I am sad to see that there are areas in the country where prevalence is as high as 40%. We should not be in a situation like this.”* (Policy maker; male).

### Opinions on the potential of current interventions for malaria elimination

Two main viewpoints were expressed regarding the potential of current interventions in leading the country to elimination by 2030. One viewpoint, expressed by a majority of participants across the stakeholder groups, was that current interventions would not be sufficient to achieve elimination, even if they were utilized fully and effectively. One key reason given was that current interventions do not address growing challenges, such as insecticide resistance, or changes in mosquito biting behaviours. As one community member explained

*“I really do not think that the insecticide*-*sprays or the bed nets are enough, because if they were enough we would not have malaria anymore. I sleep under a bed net every night, but mosquitoes still bite me when I am outside cooking or chatting with my family and friends. Sometimes I also spray my house with insecticides, but when I go inside to sleep, I see there are mosquitoes still. So then I know that these sprays are useless.”* (Community member, female).

The opposite viewpoint was also expressed, namely that currently available interventions would be enough for elimination if they were utilized to their maximum potential. As pointed out by one research scientist:

*“We already have what it takes to achieve elimination. If bed nets were properly made, properly distributed and properly used, why would we not eliminate the disease? If they killed mosquitoes as they are supposed to, if the universal distribution was actually universal, and if people actually slept under bed nets, I do not think we would need anything else…”* (Scientist, female).

Other participants pointed out that the current interventions are passive rather than active. That is, they only target female mosquitoes coming into human dwellings to feed, rather than actively targeting mosquitoes in their larval habitats and hiding places. As one policy maker stated:

*“We need means to target and eliminate all the mosquitoes, not just the ones that get inside the house. If we decide to kill mosquitoes, then we should really kill all of them. We should target them at larval stage and adult stage to make sure that we are not leaving any windows for escape.”* (Policy maker, male).

### Opinions on the need for alternative interventions for malaria elimination

There were diverse inputs from participants on the need for complementary interventions for malaria elimination in Tanzania, although a majority participants agreed that it would be necessary to complement strategies. The insights that emerged most clearly were: (a) the importance of learning from countries that have been successful in achieving elimination; (b) the importance of knowing more about current interventions, including where or why they have failed or succeeded; and (c) the need to consider combinations of interventions as a more holistic approach to achieve malaria elimination.

Those participants who emphasized the value of learning from other successful countries argued that there was no need to develop interventions from scratch, and that the country should follow in the footsteps of those who had been successful in eliminating malaria. Other participants noted that, since malaria prevalence was not homogeneous across the country, it would be essential to employ different interventions in different settings based on the specific conditions. As one participant from the regulators’ group stated:

*“Malaria prevalence is not the same in all the country. There are parts of the country that are near elimination, and there are parts that have prevalence in double digits. This should tell you that one single method is not enough for the whole country. You need to look at different places and figure out what can work where.”* (Regulator, female).

Participants who recommended combinations of interventions argued that we now have greater knowledge of mosquito behaviours than in the past, and that this knowledge can be used to target them from multiple angles to accelerate elimination. In one of the policy-makers’ FGDs, one participant noted that:

*“In order to really eliminate mosquitoes we need a combination of different strategies…We need to target all the water bodies to get rid of the larval stages, then all the hiding places like long grass and bushes, and then in the houses where they go to look for people to bite. If we do all of this, can you tell me how we can still have malaria in our country?”* (Policy-maker, male).

There were also participants who suggested that it was not wise to rush to new interventions without learning from the limitations of the current ones, and possibly addressing those first. In one session with policy-makers, a participant noted:

*“Why aren’t the bed nets killing mosquitoes? Why are the indoor insecticide sprays not killing mosquitoes? We have heard a lot about mosquitoes being resistant to the insecticides, but I still think we have not answered the question of where the resistance is coming from; what causes it and how it can be prevented or corrected. And also, do people know that the insecticides no longer kill mosquitoes? And if this is already a common knowledge, why are we still using these insecticides? I am sure that it costs a great deal of money to treat all the bed nets in the country with insecticides; but if these insecticides no longer work as insecticides, then why are we still using them?”* (Policy-maker, female).

### Opinions on the potential of alternative interventions for malaria elimination

Discussions on alternative interventions for malaria elimination were based on participants’ opinions about their effectiveness, sustainability, safety, as well as on their views on Tanzania’s readiness to adopt them. There was a wide diversity of opinions, as described below.

#### Improved housing

All stakeholder groups associated improved housing conditions with reduced malaria risk. However, there were disagreements on whether the government should support the transition towards better living conditions in malaria endemic areas. While community members were strongly supportive of this idea, policy-makers were hesitant, pointing out issues of sustainability, affordability, and competing government priorities.

Community members argued that no intervention would be fully effective without adequate housing. Specifically, they noted that none of the other interventions under discussion would be particularly useful if people continued to live in poorly-constructed houses with gaps on walls, roofs, doors and eave spaces. They further stressed that the government could indeed afford providing better housing for the poorest community members living in areas with high malaria burden. Community members proposed several ways that the government could assist, such as by providing loans for people to build improved houses, subsidizing prices for building materials, or building and renting houses to the poorest at a reduced price. As one community member said:

*“If the government could listen, I would advise them to assist people, especially the poor people, to build improved houses. They can maybe build the houses, and people can repay the government slowly, everyone can pay according to what they can afford.”* (Community member, female).

Policy-makers agreed that improved houses provide extra protection against malaria-transmitting mosquitoes. However, they were against the idea of the government building or modifying houses for poor people living in areas of high malaria transmission. They argued that it is not the responsibility of the government to build houses for citizens, and that given the required magnitude, the programme would be expensive and unsustainable. As one policy maker said:

*“You know, our country is still poor, which means that a lot more people live in poverty than not. If you say that we start building or improving houses for all the poor people, then we will not have money for any of the other important things like health care and education.”* (Policy-maker, female).

Policy-makers also indicated that building better houses alone would not be enough to eliminate malaria—a lot of effort would still be needed to ensure that mosquitoes are controlled in their larval habitats and hiding places.

Research scientists and regulators also agreed that it would be advantageous if poor people in malaria endemic areas had access to better housing. Nonetheless, they too noted that it would not be sustainable for the government to support this initiative, or even to get funding to investigate its potential. As one scientist noted:

*“For house improvement, no one denies that this works. The only problem is cost implications; that could be one of the reasons that this has not been taken up. Also, the way our research is organized and funded does not help in things like house improvement. It is difficult to get funding for [researching] this”* (Scientist, male).

#### Larval source management

Two strategies were discussed: environmental management and larviciding (Table 1). However, most of the interest was directed towards larviciding. One major issue voiced by all stakeholder groups was the lack of clear regulations and enforcement on environmental management regulations, especially in relation to settlement planning and waste water management. Community members complained about lack of regulations on where people build, cultivate crops or manufacture bricks for construction, which often results in the accumulation of standing water near settlements, increasing the risk of malaria and other mosquito-borne diseases. In the words of a community member:

“*The town is rapidly growing now. There were parts of the town that people were allowed to make bricks in the past; no one lived there at the time. But now many people live there, and it is not safe because there are so many brick*-*pits, hence so many mosquito breeding places…It would be important if there were requirements,* [for example] *that the brick makers move to other unoccupied places, or* [that] *they should be required to fill in the pits”* (Community member, female).

The use of larvicides for malaria control was perceived positively across the stakeholder groups, but with some caveats. Policy-makers strongly supported the use of bio-larvicides, stating that the government had invested on the creation of a bio-larvicide plant as part of the national strategy towards malaria elimination, but that use of the bio-larvicide remained low as one policy maker reported:

*“The bio*-*larvicides we are producing are designed to only affect mosquitoes, so they are relatively safe on the environment. We expected a high uptake from community and civil organizations, but I am sad to say that we are getting more customers from outside the country than within the country….”* (Policy-maker, female).

Research scientists were also supportive of larviciding for malaria elimination, but they noted that the efficacy of the locally produced bio-larvicides should be thoroughly evaluated, since any perception of low efficacy might cause low uptake.

While a majority of the community members were in favour of larviciding for malaria control, a few members expressed concerns that there were so many water pools in their villages, particularly in the rainy season, that it would be difficult to treat all of them with larvicides without harming the environment, particularly the fish. One person stated:

*“I would also like to stress that I do not trust this idea of putting chemicals in water. We all know that all of this water makes its way into the river where we get our fish. If we treat all the pools then that means a lot of chemicals will be going to the river. Now, are you telling me that it will not harm the fish? Most of us are fishermen here and our fish is part of who we are. Anything that can harm the fish will not be welcomed here. Maybe if you want to put these chemicals, you can do it during the dry season, but then there are not many mosquitoes during this time, so it will just be a waste”* (Community member, male).

#### Mass drug administration (MDA) of the endectocide ivermectin

Mass drug administration with ivermectin is currently undergoing trials in Tanzania as a potential vector control tool but several already completed trials suggest the potential impact of this intervention on mosquito density and malaria burden [18, 55]. When given to humans and/or cattle, the drug is effective in killing the mosquitoes that bite these hosts. The drug was widely known among all stakeholder groups as it is already widely distributed for control of lymphatic filariasis in humans [44, 45] and for several cattle diseases [56].

Community members referred to ivermectin as *Usubi*, and remembered health workers going from house to house every year encouraging people to take the drug for the control of *Matende* (elephantiasis) and *Mabusha* (hydrocele), conditions commonly associated with lymphatic filariasis. Despite the high awareness of this drug, there were mixed views among the stakeholder groups on its use for malaria control. Regulators, policy-makers and research scientists were hopeful and supportive of the approach, given its safety and effectiveness for the control and treatment of lymphatic filariasis in Tanzania. They argued that deploying it for the control of malaria-carrying mosquitoes would represent an important advantage at relatively low cost. They also stressed the need to spend time and resources to educate and raise awareness of the benefits of ivermectin use among target communities.

Community members on the other hand, had strong objections to this intervention, reporting negative experiences with previous mass drug administration (MDA) campaigns, particularly of praziquantel, commonly used for the treatment and control of schistosomiasis among school children. They reported that a number of children who receive the drug suffer fainting spells in schools. They also noted that people often avoided taking medicines. One participant stated:

*“I really must tell you that these medicines that you have to swallow have a challenge. When they brought Usubi, even with all the education and the advocacy they had provided, people still did not take the medicines. Some people just picked it so as not to make the health workers feel bad, but after they* [health workers] *left people threw the medicine away.”* (Community member, male).

#### Targeted spraying of mosquito swarms

A great deal of scepticism was expressed by all stakeholder groups about the sustainability and feasibility of targeted swarms of *Anopheles* mosquitoes with insecticide spraying. It was noted that the approach would require extensive community participation to locate the swarms, and would be expensive. One participant stated

“*The setback with this is that you need a lot of people to do that, so it may also be expensive. But I agree maybe you use less insecticides, but if you are worrying about the cost of the insecticides, you will still be spending more in paying people to spray*” (Policy-maker, male).

Community members also pointed out that it would be inconvenient to spray at the time of the day when mosquitoes swarm—around sunset—and in most of the locations where they do so: *“…it will be difficult to find someone at home during that time, people will still be at work, or they will be too tired to accept more work.”* (Community member, male).

#### Mosquito modification technologies

The possibility of releasing modified mosquitoes generated a lot of discussions and resulted in polarized viewpoints among all stakeholder groups. Although groups were introduced to different approaches to mosquito modification (i.e., sterile insect technique, genetically modified-sterile mosquitoes, and gene drive technology), most of the interest centered on implications of gene drive technologies, particularly those used for suppression of malaria vector populations.

Scientists expressed the most pointed criticisms of gene drive technology. They questioned its safety and the country’s readiness for this type of innovation. They also pointed out that there are still a lot of unknowns, and that long-term research would be needed to provide evidence on various aspects of the technology. They expressed concerns about the possibility of mutations in either the *Plasmodium* parasite or the modified mosquitoes themselves. Specific concerns in this case were that the modified malaria vectors could become vectors for other diseases, or that the parasite could mutate and survive in other mosquito species. The fact that the technology would target a single species of malaria vectors was also seen as a risk, as it could increase the prevalence or vectorial capacity of other species. Targeting one mosquito species was also seen as a drawback in securing community acceptance. One participant stated:

“*For the people, no malaria means no mosquitoes. They still cannot distinguish between malaria*-*transmitting and non*-*malaria transmitting mosquitoes, so if you tell them that you are controlling malaria then they need to see the mosquitoes gone.”* (Scientist, female).

Scientists were also concerned by the fact that there were not many African and particularly Tanzanian scientists taking leading roles in this sort of research. As one scientist stated:

*“There are more fears than certainty regarding this technology. It is mainly being driven by foreigners. I worry that there are not many African researchers participating in the detailed research of this technology”* (Scientist, female).

Policy-makers were divided in their views regarding gene drives. Some were in favour of the technology, pointing out that it was environmentally friendly and required little compliance from communities, yet others were skeptical, noting that there is currently a great deal of controversy over genetically-modified food products, and that it might, therefore, be unwise to introduce another genetically-modified organism (GMO). One policy-maker said:

“*We are already struggling with acceptance of GM crops. Adding yet something like this may bring havoc in the country. Let them [*other countries*] try it first, let us learn from our neighbours, and go last in this”* (Policy maker, female). The policy makers also recognized that the technology is not yet ready, and cannot be considered in the context of a 2030 malaria elimination target.

In contrast, and perhaps surprisingly, community members expressed a great deal of fascination with the technology. They were struck in particular by the fact that it would require little work or participation from local residents, compared to traditional malaria interventions. They also expressed a preference for this technology since it seemed to pose the least harm to the environment, particularly to fish. One participant said:

“*I like that it does not have any chemicals, so the environment and the fish are all safe, but the malaria*-*mosquitoes will be gone* (Community member, male).

Regulators pointed out that, while the potential of gene drive technologies ought to be explored, there are currently no adequate policies and regulations for their governance. Before those can be put in place, more research is needed to assure short- and long-term safety. One participant said:

*“There are regulations for GMOs, but this technology you have is not GMO, rather gene*-*edited organisms. Gene*-*edited is not the same as GMO. We do not have policies or regulations for that. I believe you [*the scientists*] can advise us on this; provide all the information needed and the evidence of its safety and we can add this into the regulations concerning GM organisms”* (Regulator, female).

#### Spatial repellents

All stakeholder groups agreed that this technology would be appropriate as a complementary (rather than primary) intervention for malaria control and elimination. Scientists however indicated that there was still insufficient evidence to determine the best spatial repellents, their availability, cost and feasibility of use.

Community members spoke positively about spatial repellents, saying they were most useful when people were outdoors in early night hours, cooking, eating and relaxing with their family and friends before going indoors to sleep. They alleged that it would be best if the government could distribute bed nets together with spatial repellents as a package, in order to tackle the problem of changes in mosquito behaviour. One participant stated:

*“We have been told that mosquitoes are clever and have changed their biting times, so we have to be smart too and respond to that change using these repellents. If the government can provide these repellents to every household and teach them when, where and how to use them, I think we can make a very big progress in ending the malaria problem.”* (Community member, female).

## Discussion

This study explored opinions of key stakeholders on Tanzania’s progress towards malaria elimination, and on the suitability and potential value of six alternative interventions that might be used to complement current efforts to achieve that goal in the future. The stakeholders weighed the pros and cons of alternative technologies for malaria control and elimination, rather than focusing their discussions on a single approach.

The findings reveal a considerable agreement across the stakeholder groups on the extent of progress achieved in the control of malaria in Tanzania over the last decade. It was also noted that policy makers, regulators and scientists pointed to statistical evidence of declining malaria prevalence, as reported in recent Tanzania’s malaria indicator surveys [3, 57], while community members pointed mostly to the lived experiences of witnessing fewer episodes of malaria and reduced severity of the disease. All participants commended the country’s efforts in providing universal coverage with LLINs, reliable diagnosis and affordable treatment, whose effectiveness has been demonstrated in various studies [7, 58, 59]. There was also a general but not unanimous agreement that current interventions will not be sufficient to achieve further reductions of the malaria burden. Participants listed various challenges, such as insecticide resistance and outdoor biting exposure, which have been registered in many field studies [5, 7].

While there was a general consensus that new, complementary interventions or technologies will be needed to push the country further towards elimination, opinions differed on what technologies deserved prioritization and investment. The interventions with broadest support were larviciding and spatial repellents. Participants favoured spatial repellents for their low cost and ability to provide temporary relief against early-evening and outdoor-biting mosquitoes, thereby complementing LLINs. Support for larviciding could be found in all stakeholders as well, and it was the most preferred option among policy makers, regulators and research scientists. Community members did not object strongly to it, but expressed concerns over its environmental impact, particularly on fish stocks, and favoured its use during the dry season to minimize the likelihood of water contamination. Current national policy already includes an expansion of larviciding as a means of achieving further reductions in malaria incidence [11].

Insecticide-spraying of mosquito swarms was the least preferred option for all stakeholder groups, due to perceived environmental harm, high cost, and the assumed difficulty of area-wide implementation and scaling up. This view contrasted with a previous survey conducted in the Ulanga and Kilombero district on the same topic, which showed wide acceptance for the targeting of mosquito swarms [60]. This difference in opinions is likely due to the fact that the community members involved in the FGDs had no real experience with the intervention, compared to the community members who had been interviewed for the survey, all of whom had volunteered in a swarm targeting trial.

One surprising outcome of this study was the degree of skepticism that scientists expressed about the prospect of mosquito modification technologies, particularly those based on gene drive constructs—and, by the same token, the comparatively positive view expressed by community members. This is a potential important observation since any introduction of gene drive-based methods for malaria control in Tanzania will require strong support by local scientists, because of basic operational reasons and the influence that scientists have on the perceptions of all the other stakeholder groups. Some of the concerns discussed by scientists, such as their doubts about safety or the possibility undesirable mutations, can be addressed by further scientific research, but others, in particular their complaint about inadequate involvement of African scientists in the development of the technology, will require changes in the social and political organization of gene drive research for the control of vector-borne diseases. Similar concerns were observed in a recent study that explored perceptions of scientists in Nigeria on the potential release of genetically modified mosquitoes [61]. In this study, policy-makers and regulators repeatedly noted they will rely on the advice of scientists to make informed decisions. This emphasizes the persuasive power of scientists, and stresses the need to expand involvement of local scientists in the development of the technology, and the need for further collaboration between scientists, policy-makers and regulators in the development and evaluation of this technology.

Community members, in contrast, expressed support for gene drive technology. They perceived it as being environmentally safer, and noted that it would require little work by communities, a welcome contrast to most current interventions. This was an unexpected finding for us, and contrasts with studies conducted elsewhere that suggest significant opposition to the release of modified mosquitoes. A recent study from Mali, for instance, reveals the reluctance of community members to accept experimental releases of genetically modified mosquitoes in their villages, arguing that the technology should be first tried elsewhere to show evidence of safety [62]. A recent US study has shown that nearly two-thirds of respondents trusted universities and the department of agriculture (but not the private sector or the Department of Defence) to develop gene drives [63]. This study further attests to the importance of gaining approval from local scientists, and the need to strengthen communication between scientists and communities in deliberating over the appropriateness of this technology. For interventions such as gene drives, this study also demonstrates that additional engagement and training for local scientists will be necessary before the intervention trials proceed.

Community members expressed a strong preference for improvement in the built environment. They emphasized that improving houses was a more sustainable approach to malaria prevention, and would have a similarly positive impact on many other vector-borne diseases. This point of view is not surprising, and is supported by historical evidence linking successes against malaria to improved housing conditions in Europe and North America [64]. They also find support in recent studies showing significant reductions in malaria transmission following improved housing materials or house screening [26, 37]. In contrast, scientists and policy makers were skeptical about investing on housing improvement as a malaria control technology, mostly because of the perceived high cost and lack of political feasibility.

Mass drug administration with ivermectin also generated polarized views among the stakeholder groups. Policy-makers, regulators and scientists expressed a strongly positive view on this technology. In contrast, it generated significant opposition from community members, who reported negative previous experiences with MDAs campaigns in primary schools for the treatment and control of schistosomiasis. This observation echoes studies conducted in Tanzania and Cameroon showing that adherence to ivermectin MDA was strongly associated with previous experiences of MDAs, even when they involved other drugs [65, 66]. Community members also pointed out that people did not generally like taking drugs, particularly when they did not suffer symptoms, which would limit the adoption of this approach.

This study had a number of limitations. Some of the interventions discussed in the FGDs were new or not very well known to some participants, and required a summary introduction by the facilitator. To minimize the influence of the facilitator on the discussion, participants were first asked to list and discuss the approaches they were familiar with, and only after they had exhausted what they knew were they offered an introduction to the other interventions. That introduction was generic; questions were often reverted back to the participants themselves to elucidate the reasons for their queries. In the discussion, an equal amount of time and information was given to each technology. Participants were generally very engaged with the discussion, and asked many questions before giving their opinion.

## Conclusion

While it seems inevitable that new tools will be needed to achieve malaria elimination in Tanzania by 2030, it remains to be seen which particular combination of interventions will be adopted in the near future. Different stakeholders perceive differently the advantages and disadvantages of each individual approach to malaria control and elimination, and assess individual options in the context of existing methods and other potential alternatives. All stakeholder groups, however, claimed that they rely on the advice provided by scientists to make informed decisions. This shows the critical role scientists play as gate-keepers for new interventions, and suggests the importance of a robust dialogue and clear communication between scientists, policy-makers, regulators and community members. Community members shared multiple thoughts on how the alternative interventions might work for them. Their willingness and in some cases eagerness to participate in efforts towards malaria elimination emphasizes the need to actively involve citizens in the design, development and implementation of strategies to eliminate malaria, in Tanzania and elsewhere. While scientists, regulators and policy-makers describe progress against malaria in terms of declining parasite prevalence, community members describe that same progress in terms of their daily life experiences. It is, therefore, vital to create an on-going dialogue between scientists, policy-makers, regulators and communities on any new interventions being considered for malaria control and elimination. Lastly, the need for local scientists to engage in development and evaluation of new technologies such as gene drives is desirable to promote uptake, should such technologies prove effective.

## Data Availability

All data for this study will be available upon request.

## Abbreviations

LLINs: Long-lasting insecticide-treated bed nets
IRS: Indoor residual sprays
LSM: Larval Source Management
FGDs: Focus group discussions
MDA: Mass drug administration
GMO: Genetically Modified Organisms
IHI: Ifakara Health Institute
NIMR: National Institute for Medical Research
